# Arthroscopy and orthopedic residency: a cross-sectional study of training structure in a resource-constrained environment

**DOI:** 10.11604/pamj.2023.45.42.36208

**Published:** 2023-05-17

**Authors:** Oladimeji Ranti Babalola, Kehinde Sunday Oluwadiya, Christian Madubueze, Ibrahim Alabi, Kenechi Madu, Aikomien Usuanlele

**Affiliations:** 1 Division of Arthroscopy and Sports Medicine, Department of Orthopaedics and Trauma Surgery, National Orthopaedic Hospital, Igbobi, Lagos, Nigeria,; 2 Department of Surgery, Ekiti State University, Ado-Ekiti, Ekiti State, Nigeria,; 3 Department of Orthopedics, National Hospital Abuja, Abuja, Nigeria,; 4 Department of Orthopedics, National Orthopaedic Hospital, Dala, Kano State, Nigeria,; 5 Department of Orthopedics, National Orthopaedic Hospital, Enugu State, Nigeria

**Keywords:** Arthroscopy, training, orthopedic residency, resources, auditing

## Abstract

**Introduction:**

orthopedic residency training was established in the West African sub-region a few decades ago, but sub-specialty in arthroscopy has only become established in the last decade. This study was aimed at evaluating available arthroscopy training resources and their impact on skill acquisition by orthopedic residents.

**Methods:**

this was a cross-sectional study involving the use of a structured online survey of consultant orthopedic surgeons and orthopedic resident doctors. Details relating to the structure of training and challenges with training and recommendations for improved training were enquired.

**Results:**

one hundred and two responses were received. There were 95% males (73) and 5% (4) females among the residents and 92% (23) males and 8% (2) females among the responding consultants. Of the residents, 47% (36) were registrars while 53% (41) were senior registrars. Seventy-six percent (77) were residents and twenty-five (24%) were consultants. Didactic lectures were the most impactful available training adjunct. Only 3% (2) of the residents had access to dry laboratory sessions with no specified number of practice hours attached. There was no computer simulation laboratory or cadaveric laboratory training facility for arthroscopy training in any of the training centres. Ninety-two percent (23) of the responding consultants would prefer a 6-12-month rotation in arthroscopy for residents. Fifty-three percent (41) of the residents had regular opportunities to participate in arthroscopic surgeries.

**Conclusion:**

orthopedic residency in arthroscopy in Nigeria is emerging and can be improved upon by increasing the available training resources and trained personnel.

## Introduction

Over the last few decades, arthroscopic surgery has grown to be an important option in the management of joint pathologies in addition to traditional open joint surgery. The pioneering works of Kenji Takagi from Japan and Eugen Bircher from Switzerland laid the foundation for this advancement in surgical practice [1,2]. Arthroscopy is a key-hole surgery of joints that uses telescopes and light source among other equipment to diagnose and treat disorders of the joint. In some centers, it is reported to constitute over a third of all orthopedic surgeries performed [3].

The practice of arthroscopy requires technical skills acquired through training. Various methods have been described to help acquire these skills. These include training in basic clinical diagnostic skills, through dry and wet laboratory sessions as well as live surgery sessions [4]. The growth of arthroscopy in Africa and indeed sub-Saharan Africa has not been without its own challenges. These challenges would include the non-availability of adequate trainers with necessary expertise, the non-availability of the required high-cost equipment necessary for patient care and live surgeries, the absence of established skills laboratories, and the long learning curve associated with the practice of arthroscopy [5].

With only a few published reports from the West African sub-region, it is important to identify and address existing challenges to advance this surgical practice in the region [6-8]. The goal of this study was to evaluate available arthroscopy training resources in our study environment, its impact on skill acquisition in orthopedic residency training and to identify the challenges confronting the training in arthroscopic surgery by resident doctors in our study environment.

## Methods

**Study design:** this was a cross-sectional study involving multiple institutions across the country involved in orthopedic residency training in Nigeria. The study was conducted between July 1^st^ 2021 and August 15^th^ 2021.

**Study setting:** the study was aimed at auditing the orthopedic residency training structure for resident doctors in Nigeria. Orthopedic residency programme for resident doctors can only be conducted in tertiary hospitals and private hospitals accredited by the National Post-graduate Medical College of Nigeria (NPMCN) and the West African College of Surgeons (WACS); the two accrediting bodies for residency training in Nigeria. Nigeria has three regional national orthopedic hospitals: one in the North-Western; another in the South-Eastern and a third in the South-Western part of the country. These centers are fully accredited for orthopedic residency training of doctors. In addition to these three, teaching hospitals, and a few private hospitals are also accredited for training. A structured online survey questionnaire designed by using an online application (surveymonkey.com USA) was the tool used in gathering the necessary data.

**Study population:** the participants were orthopedic resident doctors and orthopedic consultant surgeons with arthroscopy and sports medicine as their sub-specialty of interest. The orthopedic consultants were also members of the Arthroscopy and Sports Medicine Society of Nigeria (ASMSN).

**Sample size estimation:** the sample size for the study was determined using the convenience sampling technique based on the number of orthopedic surgeons with a special interest in arthroscopy and sports medicine and who are automatically members of the Arthroscopy and Sports Medicine Society of Nigeria (ASMSN) and orthopedic resident doctors who are members of the national association of resident doctors who volunteer to respond the survey questions.

**Variable in the questionnaire:** the content of the survey questionnaire was determined through a focused group discussion involving the authors. The survey questionnaire comprised 39 open-ended and closed questions for orthopedic residents and 24 open and closed questions for orthopedic consultants in hospitals across the country. The survey for residents included sections on the location and level of training of the respondents, training resources available, the impact on the resident's training, identified gaps in training, and suggestions on improving their training, which was an open-ended question. Those for consultant orthopedic surgeons with an interest in arthroscopy and sports medicine included sections on the location of practice, caseload of arthroscopy cases, training resources available to residents, perceived attitude of residents to utilizing the training facilities, identified gaps in training and suggestions for improving training, which also was an open-ended question.

Links to the survey questions were delivered to respondents using the WhatsApp social media platform over a 6-week period and responses were noted. Reminders were sent on a fortnightly basis to respondents who were yet to give feedback of any nature. Ethical approval was obtained from the lead investigator´s institutional health research and ethics committee.

**Data collection:** the participants were contacted through the contact information (phone numbers and e-mails) available for members of the Arthroscopy and Sports Medicine Society of Nigeria (ASMSN) for the consultants, and the orthopedic residents were contacted through their database with the National Association of Resident Doctors (NARD).

**Data collection tool:** the structured questionnaire was sent through the survey link to the participants´ mobile phone numbers and participation was completely voluntary as emphasized in the questionnaire.

**Data analysis:** descriptive statistics including frequency of responses, table, and figures were used to represent categorical variables.

**Ethical considerations:** ethical clearance was obtained from the Institutional Health Research and Ethical Committee, (Reference number; OH/90/C/IX). All respondents provided their informed consent for participation in the study. Additionally, informed consent for publication was obtained from all study participants.

## Results

**Descriptive statistics:** seventy-seven (22%) responses were obtained from 346 orthopedic residents across the country, while 25 (81%) responses were received from among 31 orthopedic consultants making a total of 102 responses. There were 95% (73) males and 5% (4) females among the residents and 92% (23) males and 8% (2) females among the responding consultants. Of the residents, 47% (36) were registrars while 53% (41) were senior registrars. Most of the respondents were practicing in either specialist orthopedic institutions or teaching hospitals (Table 1). Ninety percent of the training centers had at least partial accreditation for residency training in orthopedics by either the National Post-graduate Medical College of Nigeria (NPMC) or the West African College of Surgeons (WACS), but only 20% of the primary training institutions of the residents had well-defined subspecialties in orthopedic surgery.

**Table 1:** primary training and practice centers of respondents across hospitals in the country

Institution	Frequency
Federal Medical Centers	4
General Hospitals	2
Mission Hospitals	1
Specialist Hospitals	45
Teaching Hospitals	50

**Figure 1:**
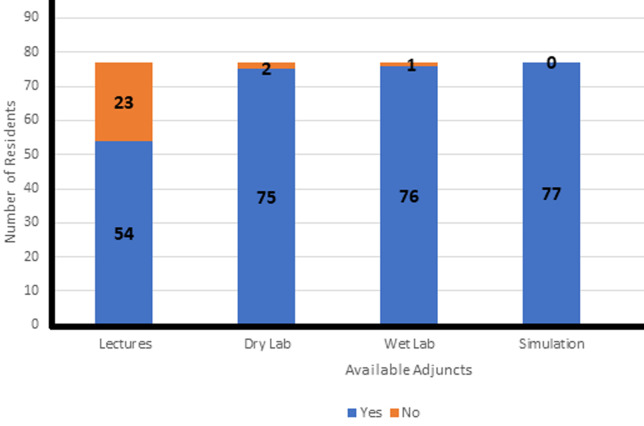
arthroscopy training adjuncts available to residents

**Available arthroscopy training resources:** most residents had no exposure to cadaveric laboratory sessions or dry laboratory sessions and none of the residents had exposure to computer simulation laboratory in arthroscopy (Figure 1). Only 3% (2) of the seventy-seven residents had access to dry laboratory sessions as an adjunct to arthroscopy training. There was no specified number of practice hours attached to the dry laboratory sessions per week in the instances where they were available.

**Figure 2:**
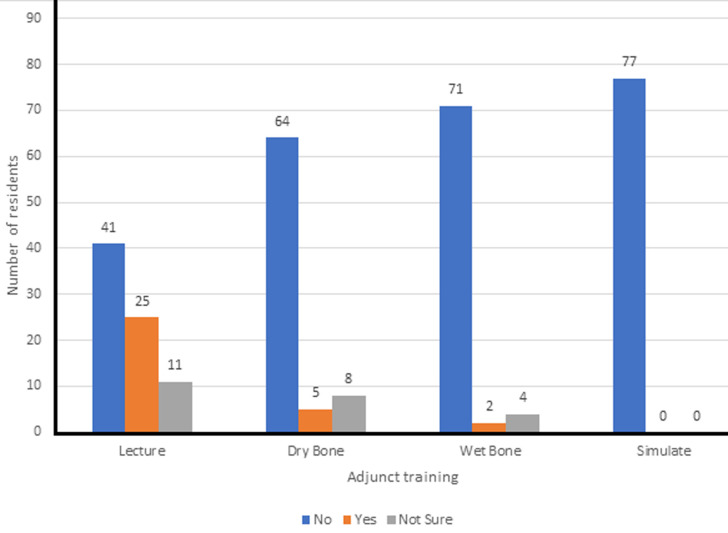
extent of impact of arthroscopy training adjuncts on residency training

**Impact of available training resources on skill acquisition by orthopedic resident doctors:** didactic lectures were reported to be the most impactful available training adjunct identified by resident doctors (Figure 2). Figure 2 highlights the fact that in the few instances where training laboratories were available, the majority of the residents did derive benefit from their sessions in these facilities.

**Figure 3:**
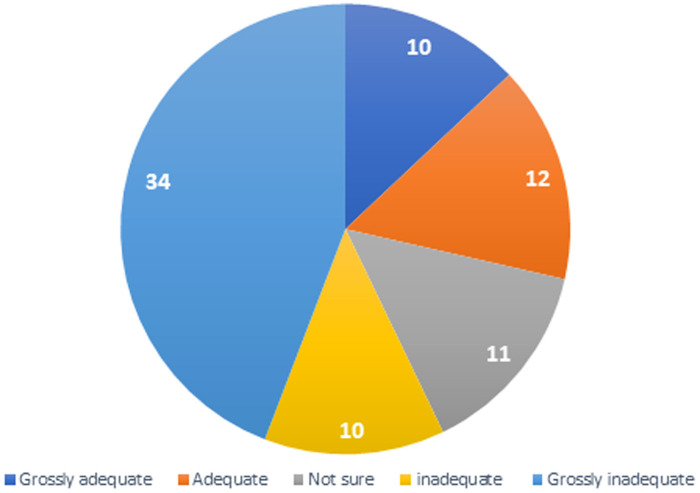
level of adequacy of arthroscopy live surgeries on gaining arthroscopy skills

Only one of these two residents could describe some of the basic arthroscopy skills expected to be gained through the dry laboratory sessions such as hand-eye coordination, triangulation, and depth perception. Five percent (4) of the residents had attended a regional basic hands-on training workshop in arthroscopy with 75% (3) of these admitting they found the workshop very useful to their training. Fifty-one percent (41) of the residents felt they had regular opportunities to participate in live arthroscopy surgeries in their training centers, but only 29% (22) admitted satisfaction with the number of cases they were exposed to seeing during rotation in arthroscopy as shown in Figure 3.

**Figure 4:**
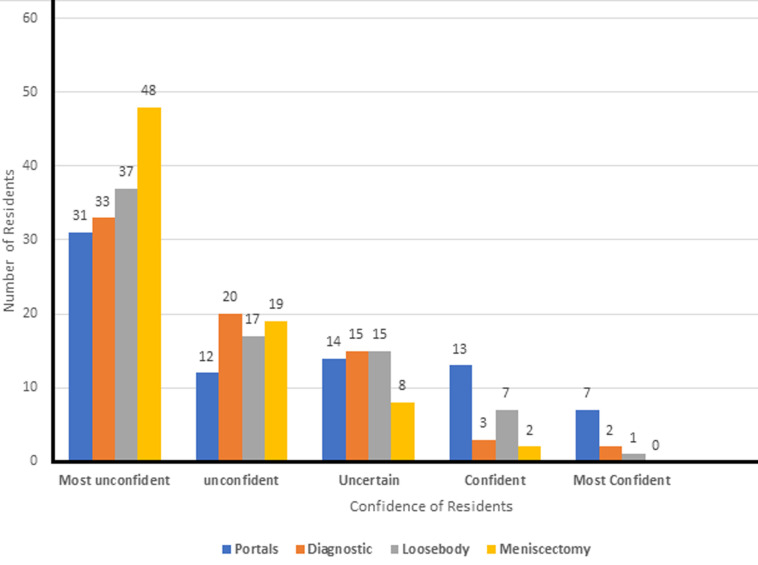
confidence of residents with performing some basic arthroscopic live surgeries in the knee joint

The impact of the live surgery sessions in acquiring arthroscopy skills among the residents was found to be inadequate as shown in Figure 4. With regard to the level of self-confidence of residents with performing some basic arthroscopic surgeries, most of the residents were not confident with performing the four referenced arthroscopic procedures (Figure 4).

**Challenges faced by orthopedic resident doctors with training in arthroscopy:** regarding the question on challenges the resident doctors encountered in their training, the responses included the following: inadequate number of arthroscopy surgeons 5% (4), lack of equipment and training facilities 91% (70), inadequate cases for clinical review and surgery 30 (39%), high cost of arthroscopy procedures 23% (18 ), very few training centers in arthroscopy in the country 30 (39%) and limited rotation time in arthroscopy 13% (10). The consultants with responses were all males. The identified challenges with the training of residents by the consultants included a shortage of consultants with an interest in arthroscopy, the high cost of arthroscopic care which discourages patients from accessing the service, lack of equipment and training laboratories, and low patient volume. Lack of equipment and training laboratories were the commonest problem noted by the arthroscopy consultants.

**Recommendations for improved training in arthroscopy:** recommendations from residents on improving the training in arthroscopy included the establishment of arthroscopy divisions in orthopedic departments in the training centers, provision of equipment such as arthroscopy carts necessary for training, structured training including wet and dry laboratory training, reduced cost of surgery, sponsored training of consultants with a keen interest in arthroscopy and better hands-on exposure.

The recommendations from orthopedic consultants involved in the training of residents included the need for the duration of rotations in arthroscopy to be 6 - 12 months in 92% (23) individuals and 3 months in 8% (2). Only 40% (10) of the consultants felt that residents rotating in the subspecialty showed keen interest in the subject. From the perspective of consultants, potential organizations that could sponsor or help facilitate the training of residents and consultants included: the Global Arthroscopy Foundation (GAF), Doctors without Borders, Surgery on Sail, European Society for Sports Traumatology Arthroscopy and Knee Arthroplasty (ESSKA), International Society for Arthroscopy Knee Surgery and Orthopedic Sports Medicine (ISAKOS).

The recommendations from the consultant for improved training also included the establishment of dedicated arthroscopy divisions in training centers, better and sponsored training opportunities for residents and consultants in better-equipped centers outside the country, provision of arthroscopy carts and other arthroscopy equipment in training centers, and availability of dry and wet laboratory facilities. Only thirteen respondents (13%) admitted to the availability of an arthroscopy cart in their practice institutions.

## Discussion

The present study is aimed at evaluating the available arthroscopy training resources in our study environment and their impact on skill acquisition in orthopedic residency training and to identify the challenges confronting the training in arthroscopic surgery by resident doctors in the study environment. Our study revealed that didactic lectures and operating room experience were the principal modes available for the training of residents in arthroscopy in Nigeria with most training centers not having adequate cases for arthroscopic surgery necessary to give residents a good exposure for their training. Additional challenges identified included the lack of equipment and training facilities, the high cost of arthroscopy procedures, and the presence of few trained arthroscopy surgeons and training centers equipped for arthroscopy in the country.

Our study showed a low level of satisfaction among residents with a caseload amenable to arthroscopic surgery. They also feel there is a low exposure to surgical skills that can be acquired from live surgeries during their arthroscopy rotations. This is reflected in the perceived low level of competence of the residents with basic arthroscopic procedures by the consultants. This emphasizes the need for greater learning opportunities outside of the operating room as the learning structure for skill acquisition in arthroscopy globally has shifted from being exclusive to the operating room to outside the operating room. Meyer R *et al*. [9] in their study similarly observed that the operating room may not be the most ideal learning environment as patient safety becomes very important in the outcome of surgery and patient satisfaction.

However, our study revealed a deficiency in the available options for “out of the operating room” training opportunities for residents in our environment. The unavailability of dry and wet laboratory training facilities, as well as computer simulation laboratories in most centers across the country, was noted in our study. Learning with these adjuncts has revealed high measures of internal validity and consistency with regard to transferring simple arthroscopy skills to the operating room [6]. These “outside the operating room” training facilities if available, would afford the residents an opportunity to acquire skills such as visual-spatial coordination and the ability to interpret three-dimensional structures as two-dimensional images with the reduced risk of iatrogenic injuries when these skills are translated to patient care during arthroscopy procedures in the operating room by residents.

Despite these challenges, the practice of arthroscopy has witnessed a steady growth in Nigeria in the last decade, creating a better awareness of the availability of the service and in turn, a steady increase in caseloads in hospitals [7,8,10]. However, as in most practice centers around the world, the experience gained by residents from live surgeries remains inadequate for the required competence necessary for residents to practice as a specialist [11-13]. The bodies involved in the training of residents in Nigeria; the National Postgraduate Medical College of Nigeria (NPMCN) and West African College of Surgeons (WACS) require 6 months of rotation in the subspecialty of arthroscopy for residents to qualify for the final fellowship examination of the respective colleges. It is assumed that the resident would have had the opportunity to see enough cases and have a good hands-on experience over this period. The Residency Review Committee for the Accreditation Council of Graduate Medical Education (ACGME) in the USA does not specify the required case volume for residents rotating through arthroscopy [14]. In both scenarios, no specification was given to the minimum number and type of cases an arthroscopy resident must observe or participate in managing to be eligible for the final fellowship examination.

The major challenges with the training of residents identified from our study were the lack of equipment and training facilities, inadequate cases for clinical review and surgery, high cost of arthroscopy procedures, and the presence of few trained arthroscopy surgeons and training centers equipped for arthroscopy in the country. This, coupled with the huge financial outlay needed to set up and manage cadaveric laboratories and computer simulation laboratories may be prohibitive in a resource-constrained setting such as ours [15].

From the foregoing, recommendations to improve arthroscopy training in the country would include the need for local and international partnerships between centers within and outside the country to help build capacity in arthroscopy. This would entail international health institutions with high case volumes offering student exchange programmes, scholarships, and fellowship opportunities to low-volume centers such as ours. Additionally, there is a need for international and regional centers with established arthroscopy practices to support centers like ours with low-cost arthroscopy equipment, ensuring that orthopedic resident doctors receive adequate training in arthroscopy. Furthermore, centers in Nigeria would benefit from donations of low-cost or refurbished arthroscopy equipment, reading materials, and instruments from philanthropic organizations, local and regional governments in the country, and industry partners. Also, support from state and regional government authorities to fund the training of resident doctors and consultants would positively impact arthroscopy practice in the country.

One of the limitations of this study is the use of an online questionnaire, which may be subject to response bias and incomplete or incorrect information from participants. The study's cross-sectional design, which only captures a snapshot of the situation at one time, also prevents us from inferring causal relationships between the availability of resources and the acquisition of skills. The possible impact of additional contextual factors, such as institutional funding and policies, which may also have an impact on the standard of arthroscopy training was not examined by the study. Despite these drawbacks, the study has several advantages, which include its emphasis on a portion of orthopedic residency training that has received little attention. This focus offers insightful information about the state of arthroscopy training resources in the study area and how they affect the acquisition of skills. The study gains a thorough understanding of the training landscape from various perspectives by including both consultant orthopedic surgeons and orthopedic resident doctors as respondents. The data was gathered from a wide variety of training facilities within the study area with the use of an online questionnaire. Additionally, the questionnaire's inclusion of both closed- and open-ended questions enable a more thorough analysis of the difficulties and suggestions for enhancing arthroscopy training in Nigeria.

## Conclusion

Our study has shown, from both the trainers´ and the trainees´ perspectives, that arthroscopy residency training in Nigeria is limited due to the unavailability of relevant training equipment and surgical instruments, as well as a low caseload of patients who can afford the arthroscopy surgery required to treat their disease condition.

### What is known about this topic

- *There is a very low coverage and in certain instances unavailability of technologically driven quality health care in several low- and middle-income countries;*
- *Volunteer international medical outreaches may help bridge this gap;*
- *Arthroscopic surgery is equipment driven and can be costly*.

### What this study adds

- *Arthroscopy residency training and facilities in Nigeria is presently at the very early stages of development;*
- *There is a dearth of training equipment and trained personnel in the country;*
- *A lot more improvement can be gained with the availability of training resources and trained personnel through local and international collaborations*.
